# Prioritization of landscape connectivity for the conservation of Peary caribou

**DOI:** 10.1002/ece3.4915

**Published:** 2019-01-24

**Authors:** Conor D. Mallory, Mark S. Boyce

**Affiliations:** ^1^ Department of Biological Sciences University of Alberta Edmonton Alberta Canada; ^2^ Department of Environment Government of Nunavut Iglulik Nunavut Canada

**Keywords:** climate change, connectivity, metapopulation, Peary caribou, *Rangifer tarandus pearyi*, sea ice

## Abstract

Adequate connectivity between discontinuous habitat patches is crucial for the persistence of metapopulations across space and time. Loss of landscape connectivity is often a direct result of fragmentation caused by human activities but also can be caused indirectly through anthropogenic climate change. Peary caribou (*Rangifer tarandus pearyi*) are widely dispersed across the islands of the Canadian Arctic Archipelago and rely on sea ice to move seasonally between island habitats throughout their range. Seasonal connectivity provided by sea ice is necessary to maintain genetic diversity and to facilitate dispersal and recolonization of areas from which caribou have been extirpated. We used least‐cost path analysis and circuit theory to model connectivity across Peary caribou range, and future climate projections to investigate how this connectivity might be affected by a warming climate. Further, we used measures of current flow centrality to estimate the role of High Arctic islands in maintaining connectivity between Peary caribou populations and to identify and prioritize those islands and linkages most important for conservation. Our results suggest that the Bathurst Island complex plays a critical role in facilitating connectivity between Peary caribou populations. Large islands, including Banks, Victoria, and Ellesmere have limited roles in connecting Peary caribou. Without rigorous greenhouse gas emission reductions our projections indicate that by 2100 all connectivity between the more southern Peary caribou populations will be lost for important spring and early‐winter movement periods. Continued connectivity across the Canadian Arctic Archipelago, and possibly Peary caribou persistence, ultimately hinges on global commitments to limit climate change. Our research highlights priority areas where, in addition to emission reductions, conservation efforts to maintain connectivity would be most effective.

## 1 INTRODUCTION

Maintaining and restoring connectivity between isolated patches of suitable habitat on heterogeneous landscapes has been a topic of considerable research in ecology for well over three decades (Fahrig & Merriam, 1985, 1994; Kindlmann & Burel, 2008; Opdam & Wascher, 2004; Saunders, Hobbs, & Margules, 1991). Indeed, sufficient connectivity among habitat patches enables a variety of behaviors integral to long‐term population persistence, from finer‐scale movements between patches by individuals for foraging (FitzGibbon, Putland, & Goldizen, 2007; Frey‐Ehrenbold, Bontadina, Arlettaz, & Obrist, 2013; Henry, Pons, & Cosson, 2007), to larger‐scale movements related to dispersal, reproduction, and migration (McClure, Hansen, & Inman, 2016; Rabasa, Gutiérrez, & Escudero, 2007; Rabinowitz & Zeller, 2010). Furthermore, at broader temporal scales, connectivity facilitates colonization and recolonization of ranges (Franken & Hik, 2004; Hanski, 1998) and gene flow between populations (Holderegger & Wagner, 2008), which in turn determines potential for genetic differentiation, inbreeding depression, local adaptation, and the geographic spread of novel adaptations (Keyghobadi, Roland, & Strobeck, 2005).

Recent interests in connectivity in conservation biology are largely driven by increased habitat fragmentation and habitat loss associated with anthropogenic activities such as forestry, agriculture, and urban development (Cushman, 2006; Fischer & Lindenmayer, 2007; Haddad et al., 2015; Haila, 2002). However, anthropogenic activities also can have important indirect effects on connectivity through intermediate mechanisms such as climate change (Heller & Zavaleta, 2009). In particular, changing sea ice conditions are predicted to have large implications for some Arctic species (Post et al., 2013), including caribou (*Rangifer tarandus*), Arctic fox (*Vulpes lagopus*), and Arctic wolf (*Canis lupus*), that use sea ice to move between island habitats across the High Arctic (Carmichael et al., 2008; Jenkins et al., 2016; Mallory & Boyce, 2018; Miller, Barry, & Calvert, 2005; Miller, Russell, & Gunn, 1977; Norén et al., 2011; Poole, Gunn, Patterson, & Dumond, 2010). Sea ice cover has declined at a faster rate than anticipated by many studies (Comiso, Parkinson, Gersten, & Stock, 2008; Stroeve et al., 2012), and projections of future Arctic ice loss warrant continued attention within conservation biology (Overland & Wang, 2013). Understanding how declining sea ice coverage will affect connectivity is necessary to anticipate, and potentially mitigate, some negative consequences of climate change for these species.

Peary caribou (*R. t. pearyi*) is a subspecies of caribou that resides in the Canadian High Arctic near the northern limit of vegetation growth (Miller & Gunn, 2003a). Characterized by their small stature (approximately 90 cm at the shoulder), Peary caribou live at low densities and move seasonally between Arctic islands to forage across areas of higher productivity, a behavior that also could reduce pressure on limited forage resources (Miller et al., 1977). Between‐island movements also might involve attempts to avoid predators (Miller, 2002) and to move away from areas that have been subject to extreme weather or icing events (Jenkins et al., 2016; NWT Species at Risk Committee, 2012). Although caribou typically dig through snow to access vegetation in a behavior called cratering (Fancy & White, 1985), they are unable to dig through basal layers of ice, which can lead to starvation (Tyler, 2010). Mass starvation of Peary caribou (Miller & Barry, 2009; Miller & Gunn, 2003a) and Svalbard reindeer (*R. t. platyrhynchus*) (Hansen, Aanes, Herfindal, Kohler, & Sæther, 2011; Tyler, 2010) following severe snow and icing events are well documented, and sea ice that allows animals to move away from areas where forage has been rendered inaccessible might help caribou avoid starvation (Loe et al., 2016).

**Figure 1:**
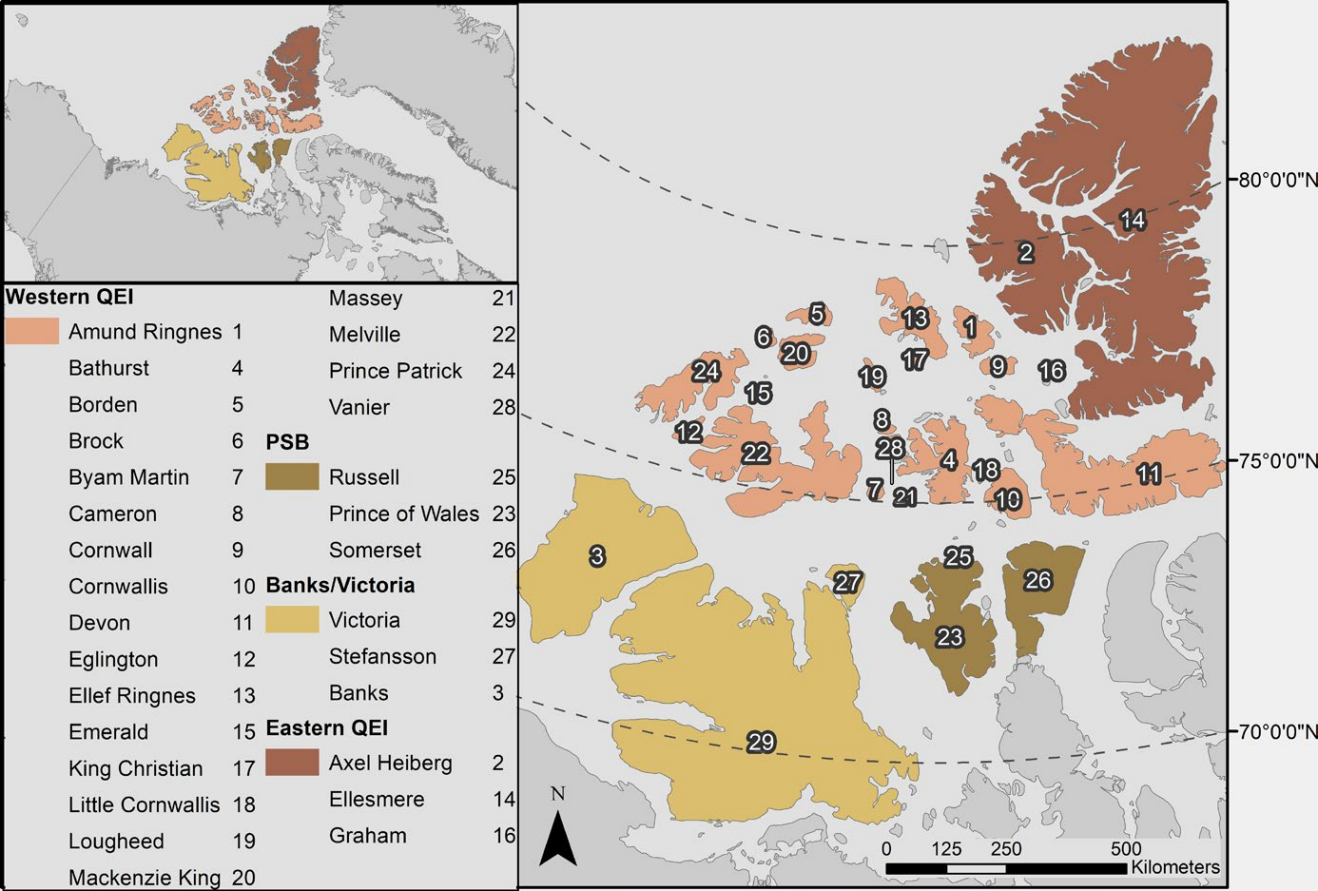
Study area and Peary caribou (*Rangifer tarandus pearyi*) local populations within the Canadian Arctic Archipelago. Islands with gray fill were not considered in our analysis. Inset map shows study area location within Canada

The delineation of Peary caribou populations used by the Committee on the Status of Endangered Wildlife in Canada (COSEWIC, 2015) and Environment and Climate Change Canada (ECCC) (Johnson, Neave, Blukacz‐Richards, Banks, & Quesnelle, 2016) comprises four local populations named for their geographic areas: Banks/Victoria Islands (BV), Prince of Wales/Somerset/Boothia (PSB), Western Queen Elizabeth Islands (WQEI), and Eastern Queen Elizabeth Islands (EQEI) (Figure 1). A large decline in Peary caribou numbers across their range, in part due to catastrophic die‐offs related to extreme snow and icing events, led to their listing as endangered under the Canadian *Species at Risk Act *in 2011. More recently in 2015, COSEWIC assessed Peary caribou as threatened in light of increasing or stable population trends in three of four local populations (COSEWIC, 2015). The most recent surveys addressing the PSB population occurred in 2004, 2006, and 2016 (Anderson, 2016; Gunn, Miller, Barry, & Buchan, 2006; Jenkins, Campbell, Hope, Goorts, & McLoughlin, 2011) and recorded only a single caribou among all three surveys. While observations of small numbers of caribou have been reported by local people (Anderson, 2016), this population appears to be near extirpation (Johnson et al., 2016).

Genetic evidence suggests that sea ice has historically facilitated reliable and effective connectivity between Peary caribou populations (Jenkins et al., 2016; Jenkins, Yannic, Schaefer, Conolly, & Lecomte, 2018). However, work by Jenkins et al. (2016) identified that projected longer ice‐free seasons across the Arctic are likely to reduce connectivity between caribou populations restricted to islands, leading to increased genetic and demographic isolation. Building on these findings, our objective is to identify areas of Peary caribou habitat that contribute most to maintaining connectivity during annual periods important for movement, and project how this might change in the future. Specifically, we used future climate projections, circuit theory, and least‐cost models to: (a) map connectivity across the Canadian Arctic Archipelago, (b) determine how specific connections might be altered under climate change scenarios, and (c) identify those areas which contribute most to maintaining connectivity across Peary caribou populations.

## 2 MATERIALS AND METHODS

To investigate landscape connectivity across Peary caribou range, we employed methods from circuit theory and least‐cost path (LCP) analysis. Both methods represent the landscape as a surface on which different habitat types are assigned different a priori resistance values reflecting ecological constraints to movement (McRae, Dickson, Keitt, & Shah, 2008). LCP analysis identifies the optimal path between two locations in terms of lowest “cost” or “resistance” (Adriaensen et al., 2003), whereas circuit theoretic approaches consider the flow of current through multiple alternative pathways across a continuous surface. Aspects of electrical circuits can be directly related to random walks, providing a straightforward link to movement ecology, with current flow interpreted as the “expected net movement probabilities of random walkers moving through a node…” or cell (McRae et al., 2008). Measures of network centrality, which evaluate the contribution of habitat nodes in facilitating ecological flows across the landscape, also can be derived from landscape connectivity models. By considering paths between all nodes, centrality metrics rank the importance of individual habitat nodes to maintaining connectivity throughout the network. For example, a node through which many paths in the network pass would have a higher centrality score than a node through which only a few paths pass (Carroll, McRae, & Brookes, 2012). Centrality provides an analytical method by which conservation priorities for maintaining connectivity can be identified (e.g., Dutta, Sharma, McRae, Roy, & DeFries, 2016; Theobald, Reed, Fields, & Soulé, 2012; Osipova et al., 2018).

### 2.1 Study area

We analyzed connectivity among 29 islands in the Canadian Arctic Archipelago (Figure 1). Our study area stretches approximately from 126°W to 61°W and 68°N to 82°N, from the Beaufort Sea in the west to Greenland and Baffin Bay in the east, and from the Canadian mainland in the south to the Arctic Ocean in the north. We excluded sections of the archipelago that are not Peary caribou range (e.g., Baffin and Bylot Islands). Characteristics of islands and habitats considered in our connectivity analysis vary considerably. Island area ranges from approximately 450 km^2^ to 220,000 km^2^ (Massey and Victoria Islands, respectively). Habitats vary from areas of graminoid tundra at the southern extent of the study area to regions of sparse vegetation and barren polar desert as latitude increases (Gould, Raynolds, & Walker, 2003; Olthof, Latifovic, & Pouliot, 2008). There is also substantial variability in productivity within some islands and latitudes, such as in Polar Bear Pass (Nanuit Itillinga) National Wildlife Area on Bathurst Island, and on the Fosheim Peninsula and Lake Hazen regions of Ellesmere Island. These “High Arctic oases” have much higher productivity and species diversity than typically found across the archipelago (France, 1993; Michelutti, McCleary, Douglas, & Smol, 2013; Sheard & Geale, 1983).

### 2.2 Data sources

We defined two seasons to investigate changes in habitat connectivity, an early‐winter season (November–December) and a spring season (April–June). These seasons were chosen due to their importance for movement between island habitats. Although movement data are limited, spring migration has been recorded in April–June, while early‐winter movements often begin in late October or November (Gunn & Dragon, 2002; Johnson et al., 2016). Sea ice concentration (SIC) projections were taken from the output of the Canadian Regional Climate Model (CanRCM4) produced by the Canadian Centre for Climate Modelling and Analysis (Scinocca et al., 2016). SIC estimates the percent coverage of sea ice within each 25‐km grid cell. Our analysis considered three scenarios: a recent historical climate scenario (1991–2005), the Representative Concentration Pathway (RCP) 4.5 scenario, and the RCP 8.5 scenario. The RCP scenarios represent projected atmospheric composition under different greenhouse gas (GHG) emission regimes (Meinshausen et al., 2011; van Vuuren et al., 2011). The RCP 8.5 scenario projects increasing GHG emissions beyond 2100, while the RCP 4.5 scenario reflects a more moderate trajectory with emissions reaching a maximum around 2040 and declining thereafter (Meinshausen et al., 2011).

Monthly values for SIC were retrieved for the historical climate scenario from 1991 to 2005, and for the RCP 4.5 and RCP 8.5 scenarios from the year 2021 to 2100. For the winter season, we considered SIC in the months of November and December, and for the spring season, SIC in April, May, and June. For each grid cell in the study area, we calculated the mean SIC value across these months for both seasons for each year. Annual seasonal means were then collected into decadal groups (e.g., 2021–2030, 2031–2040, 2091–2100), and the mean SIC for each grid cell was calculated for both seasons in each decade.

We used Peary caribou habitat models developed by ECCC to inform the terrestrial portions of our connectivity analysis. A complete description of the modeling approach and results can be found in Johnson et al. (2016), and here, we only provide a brief discussion to highlight the information necessary for our study. Peary caribou seasonal habitat use models were developed using Maxent (Phillips, Anderson, & Schapire, 2006). Habitat use was modeled for three seasons, April to June, July to October, and November to March. Known caribou locations were derived from surveys, radio‐collared animals, and information from communities on Peary caribou distribution (Johnson et al., 2016). The models relate these known locations to environmental predictor variables, including snow depth, land cover, and wind speed. From these models, ECCC produced relative probability of use by Peary caribou for the Canadian Arctic Archipelago at 1‐km resolution. We resampled the relative probability of use data from the April to June and November to March models to the same resolution (25 km) as the SIC data for use in our spring and winter connectivity analysis.

### 2.3 Landscape resistance

Estimates of landscape resistance were derived from two sources. We reclassified the spring and winter probability of use rasters into 10 bins as shown in Table 1. Rather than transform probability of use values into resistance by some function, we chose to bin ranges of probability values to reflect the variation that was lost by resampling probabilities from 1‐ to 25‐km grid cells. The classification bin with the highest probability of use was given a resistance value of 1, and the value of each successive bin was increased by 1. The two bins with the lowest probability of use (approximately 0%–15%) were given resistance values of 20 and 30. We greatly increased the value of these lowest two bins so that in habitats or on islands where probability of use was very small, caribou would be more likely to move onto or across the sea ice than through those habitats.

**Table 1:** Terrestrial landscape resistance values derived from the reclassification of Peary caribou (*Rangifer tarandus pearyi*) probability of use across the Canadian Arctic Archipelago

Resistance	Probability of use	
	Spring	Winter
1	0.73–0.83	0.76–0.88
2	0.65–0.73	0.64–0.76
3	0.58–0.65	0.55–0.64
4	0.50–0.58	0.48–0.55
5	0.41–0.50	0.39–0.48
6	0.33–0.41	0.30–0.39
7	0.24–0.33	0.23–0.30
8	0.16–0.24	0.15–0.23
20	0.09–0.16	0.07–0.15
30	0.00–0.09	0.00–0.07

Resistance values for sea ice were derived from the SIC for each 25 × 25 km cell. SIC was transformed to a resistance value by multiplying SIC by −1 and then adding 100, such that high SIC values were given low resistance scores. SIC values less than 70 were set to null (infinite resistance) to render them impassable for our connectivity analysis, and 10 was added to all remaining values (i.e., a grid cell with SIC 99 would be assigned resistance 11, and a SIC grid cell with SIC 70 would have resistance 40). Values were shifted by 10 so that in our analysis, unless a terrestrial grid cell has very low probability of use (i.e., the 20 or 30 resistance bins) moving onto or across sea ice presents higher resistance to caribou than traveling on land. Previous work has suggested that caribou require at least 90% ice coverage to make crossings (Poole et al., 2010), and in their connectivity analysis, Jenkins et al. (2016) differentiated resistance only between sea ice and ice‐free waters. Although our use of 70% ice coverage for caribou to make crossings is likely unrealistic at a finer scale, the coarse resolution of our analysis and potential variability of ice conditions within a 25 × 25 km cell necessitated a lower cutoff, particularly in near‐shore areas where a grid cell contains both ocean and land.

Assigning values to a resistance surface often involves some subjectivity (Spear, Balkenhol, Fortin, Mcrae, & Scribner, 2010; Zeller, McGarigal, & Whiteley, 2012), and our study is no exception. However, because we focus on the changes to connectivity resulting from complete loss of permeability of a grid cell (i.e., SIC <70%) our analysis is relatively robust to the specific resistance values of individual grid cells. So long as the rank order of grid cells derived from the probability of use analysis remains constant, changing resistance scores will affect the absolute values returned by our analysis, but should have little effect on the overall patterns we report.

### 2.4 Connectivity analysis

We used Linkage Mapper (McRae & Kavanagh, 2011), Circuitscape (McRae, Shah, & Mohapatra, 2013), Centrality Mapper (McRae, 2012), and ArcMap 10.4.1 (Esri, 2015) to investigate changes in connectivity across Peary caribou habitat. These programs provide methods which combine circuit theory with LCP analysis. To analyze connectivity across the study area, we used Circuitscape to iteratively calculate current flow across all possible pairs of islands (McRae et al., 2013). For each pair, one amp of current was injected into one of the islands while the other was connected to ground. For each calculation, the islands of the focal pair were treated as homogenous regions of zero resistance, but all other regions maintained their assigned resistance values. The current densities from each calculation were then summed to produce maps of cumulative current density across the study area for each decade and climate scenario.

We then used Linkage Mapper to identify and construct a network across adjacent core areas (in our case islands of the archipelago). Linkage Mapper then calculated cost‐weighted distances and LCPs between islands (core areas) and produced a map of the resulting least‐cost corridors. After corridors were mapped we ran Centrality Mapper, which uses circuit theory (through Circuitscape) to calculate current flow centrality across the nodes and linkages of the LCP network. Centrality Mapper treats each island as a node and links between nodes are given a resistance value derived from the cost‐weighted distance of each particular least‐cost corridor. Core areas are paired, and the program injects current into one core area while setting the other to ground. Centrality Mapper iterates over all pairs and sums the resulting current for all nodes and links in the network. We standardized the resulting centrality values for each island by dividing the centrality value by the island's area.

To estimate projected changes in landscape connectivity across Peary caribou range, we compared current density and the number of linkages between islands for each decade from 2020 to 2100. To identify which islands contribute most to maintaining landscape connectivity, we evaluated current flow centrality for all islands for each climate scenario and decade. We ran these analyses for all decades from 2020 to 2100, but to be concise we report a representative subset here to illustrate our findings.

## 3 RESULTS

**Figure 2:**
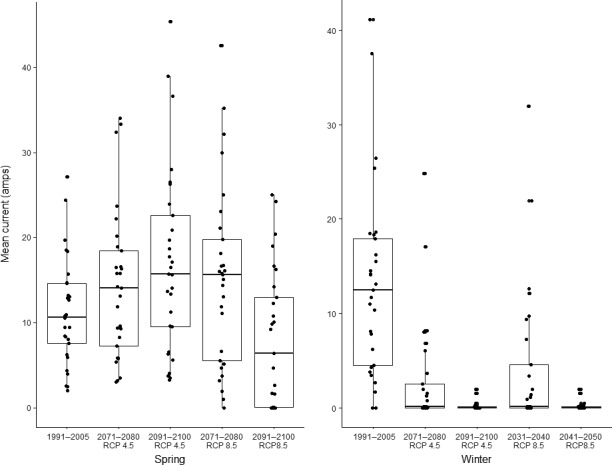
Mean cumulative current density for study islands in the Canadian Arctic Archipelago grouped by decade and climate scenario. Each point represents the mean cumulative current passing through the cells of a given island

**Figure 3:**
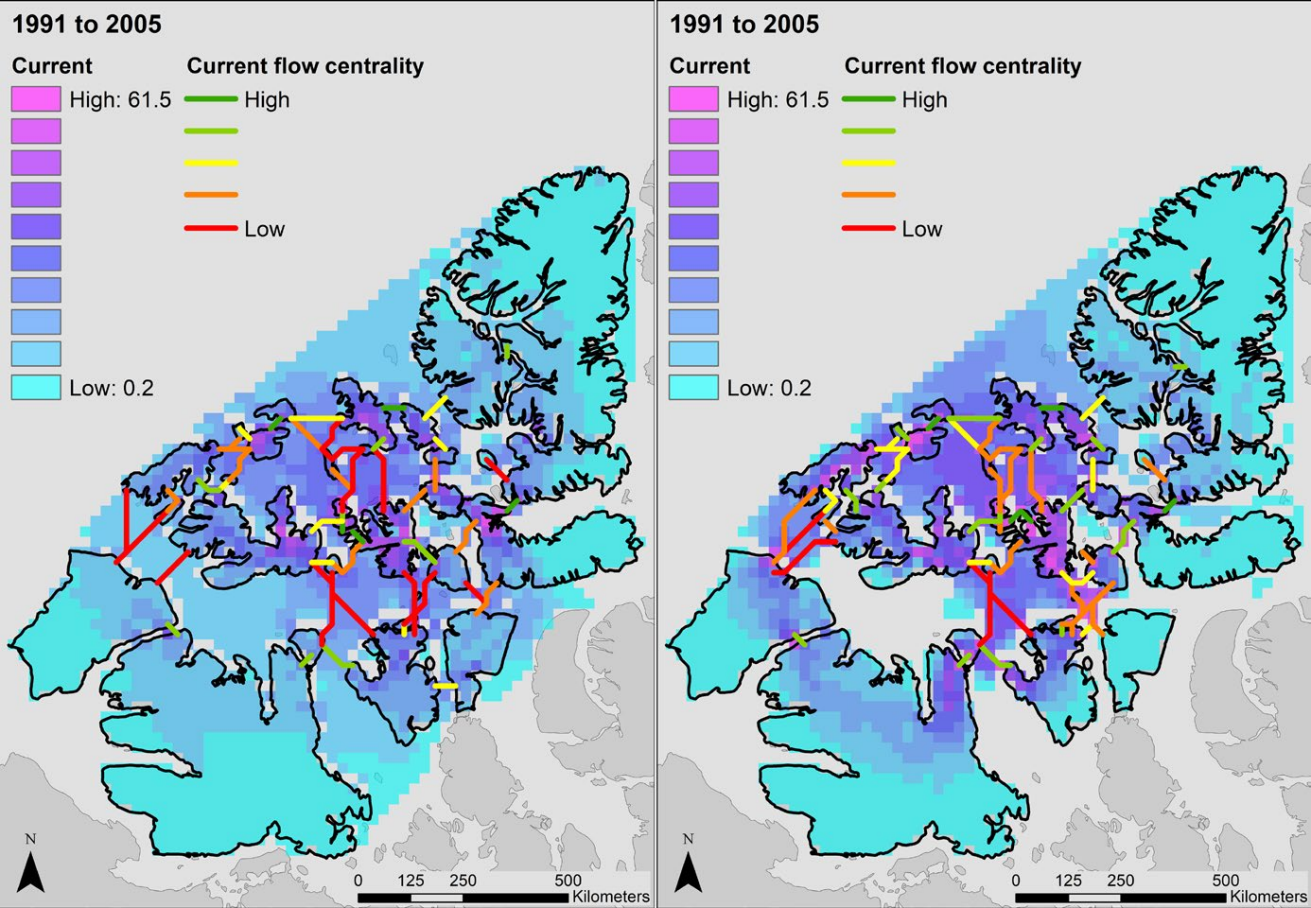
Cumulative current density (amps) across the study area in the Canadian Arctic Archipelago in spring (left) and winter (right) under the historical (1991–2005) Canadian Regional Climate Model (CanRCM4) scenario. Cumulative current density can be interpreted to reflect movement probabilities of Peary caribou (*Rangifer tarandus pearyi*)

**Figure 4:**
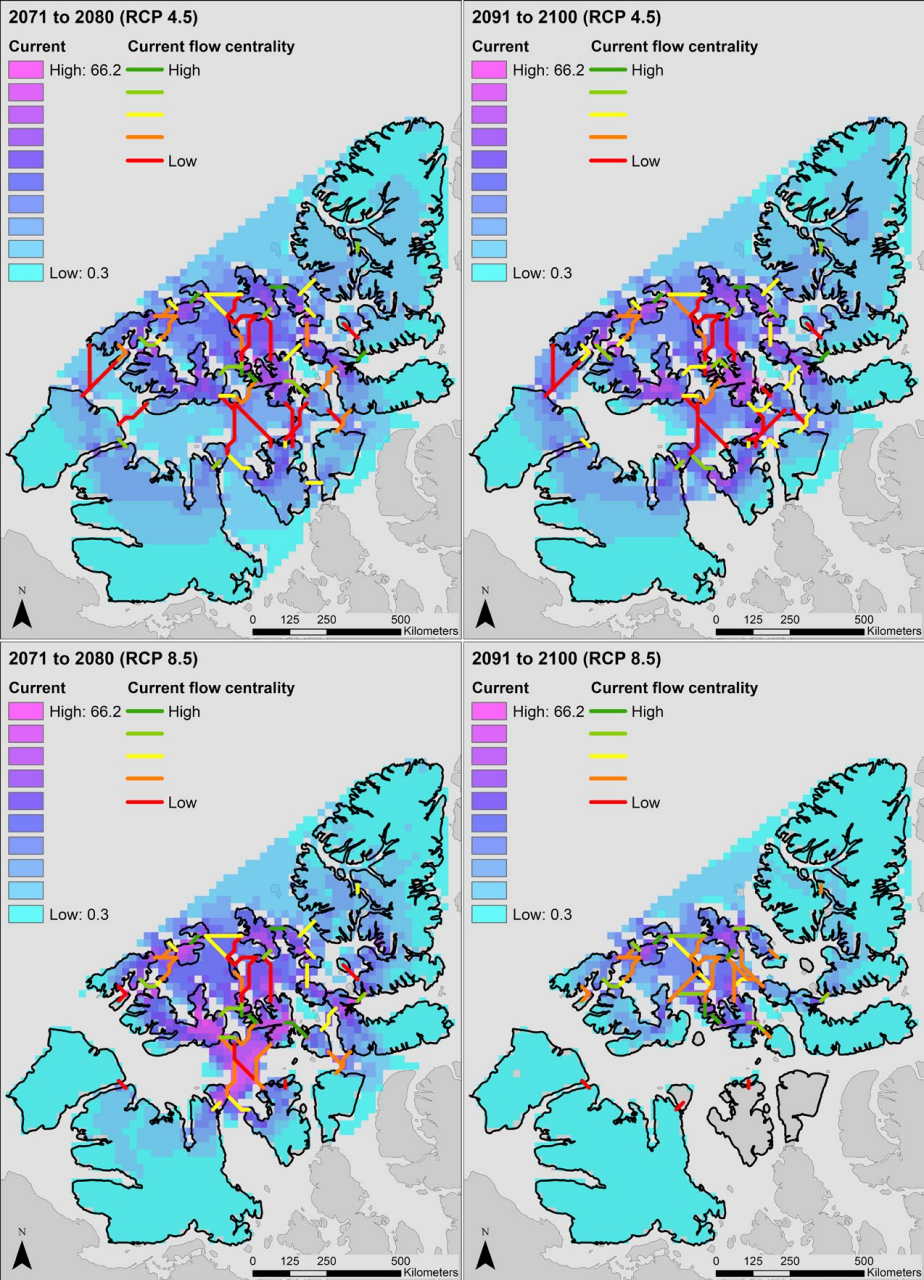
Cumulative current density (amps) across the study area in the Canadian Arctic Archipelago in spring under RCP 4.5 (top) and RCP 8.5 (bottom) projections from the Canadian Regional Climate Model (CanRCM4). Cumulative current density can be interpreted to reflect movement probabilities of Peary caribou (*Rangifer tarandus pearyi*)

**Figure 5:**
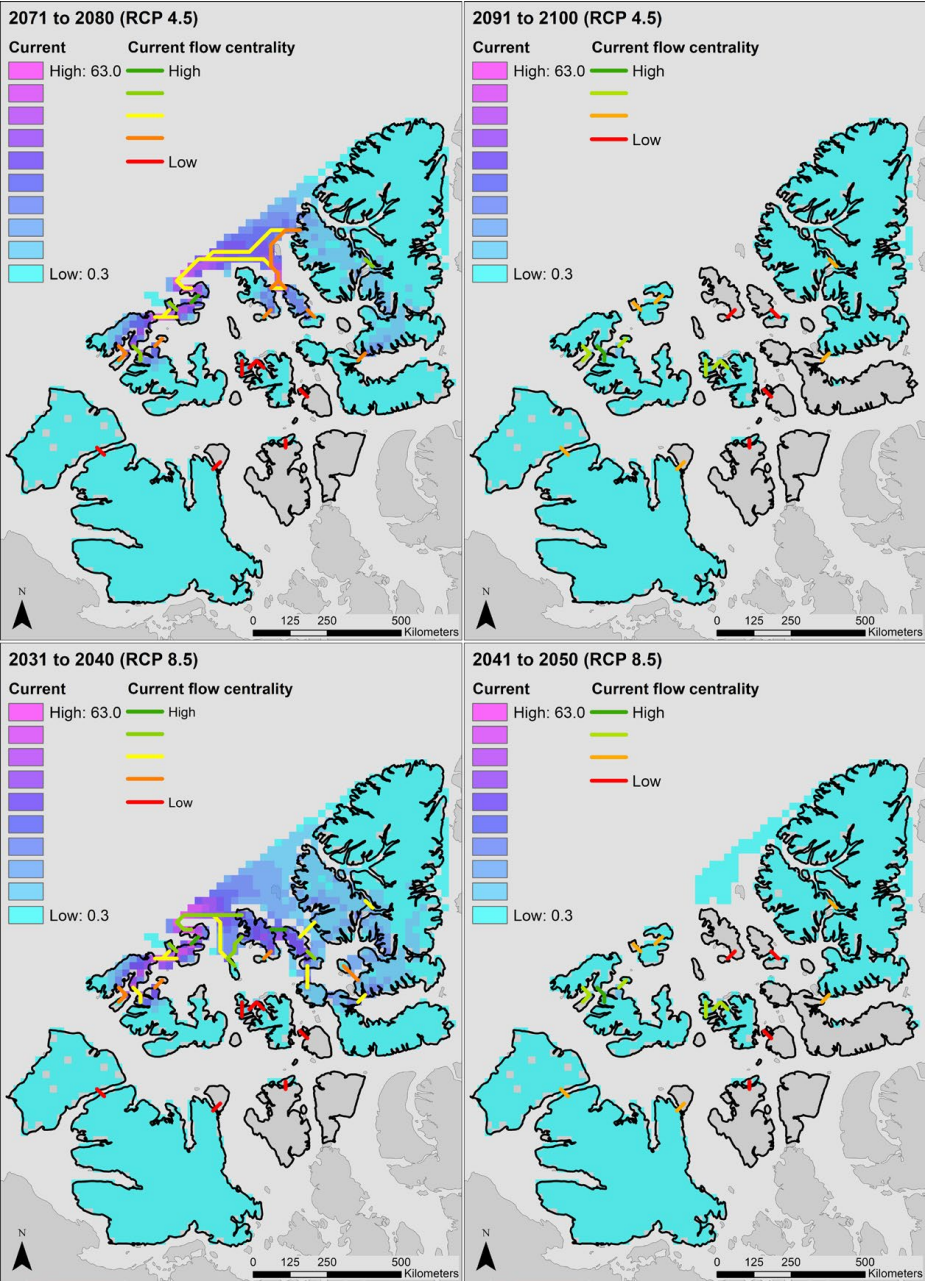
Cumulative current density (amps) across the study area in the Canadian Arctic Archipelago in early winter under RCP 4.5 (top) and RCP 8.5 (bottom) projections from the Canadian Regional Climate Model (CanRCM4). Cumulative current density can be interpreted to reflect movement probabilities of Peary caribou (*Rangifer tarandus pearyi*). Note that the time periods shown here are not the same as in Figure 4

Our analysis indicates that a longer ice‐free season in the Canadian High Arctic will dramatically decrease connectivity between Peary caribou island habitats during important movement periods in both winter and spring. In Figure 2, we display changes in cumulative current density under the RCP 4.5 and 8.5 scenarios. In spring, modeled cumulative current density under the RCP 4.5 scenario increased as the loss of sea ice and higher resistance of remaining ice reduced the number of paths current could take across the study area, leading to increased current flow across some islands. Under the spring RCP 8.5 scenario, a more rapid loss of sea ice resulted in more variable changes in modeled cumulative current. Current density increased for some islands until 2080 as occurred under the more moderate scenario (e.g., Bathurst, Mackenzie King), but declined by 2100 as SIC continued to decrease and connectivity was lost. In other cases, the complete loss of connectivity between islands leads to reduced cumulative current density by 2080 (e.g., Victoria and Somerset Islands). Under the RCP 4.5 scenario, mean cumulative current density on land increased by approximately 27% by 2080, and 51% by 2100. Under the RCP 8.5 scenario, mean cumulative current density increased 35% by 2080 but declined 29% from its historical value by 2100. For the winter period, the faster rate of sea ice loss resulted in large reduction in cumulative current density across the study area. Under the RCP 4.5 scenario, mean cumulative current density declined by approximately 78% by 2080, and 99% by 2100. Under the RCP 8.5 scenario, declines are sharper, with a 68% loss in cumulative current density by 2040 and 99% by 2050. Changes in cumulative current flow are represented spatially in Figures 3, 4, and 5.

### 3.1 Least‐cost paths

Loss of connectivity was projected to be severe during the early‐winter period, when all connectivity between islands further apart than the minimum resolution of our analysis (25 km) was lost under the RCP 8.5 scenario by 2050. Under the RCP 4.5 scenario, other than between some WQEI, all early‐winter connectivity was lost by 2100. In spring, all between‐population connections (e.g., BV‐WQEI, WQEI‐PSB) were lost by 2100 under the RCP 8.5 scenario. The number of modeled connections between islands dropped from 49 to 33 (33%) by 2100, and all remaining connections were between WQEI. Loss of spring connectivity under the RCP 4.5 scenario was less severe, with connections between populations maintained. Under the spring RCP 4.5 scenario, only two connections were lost, from 49 to 47. In winter, connections decreased from 49 to 25 (49%) by 2040 under the RCP 8.5 scenario, and by 2050, all connections of greater distance than the minimum resolution were lost. Under the winter RCP 4.5 scenario, connections declined from 49 to 16 (67%) by 2100. The 16 remaining connections were between WQEI (Figure 4).

### 3.2 Current flow centrality

**Figure 6:**
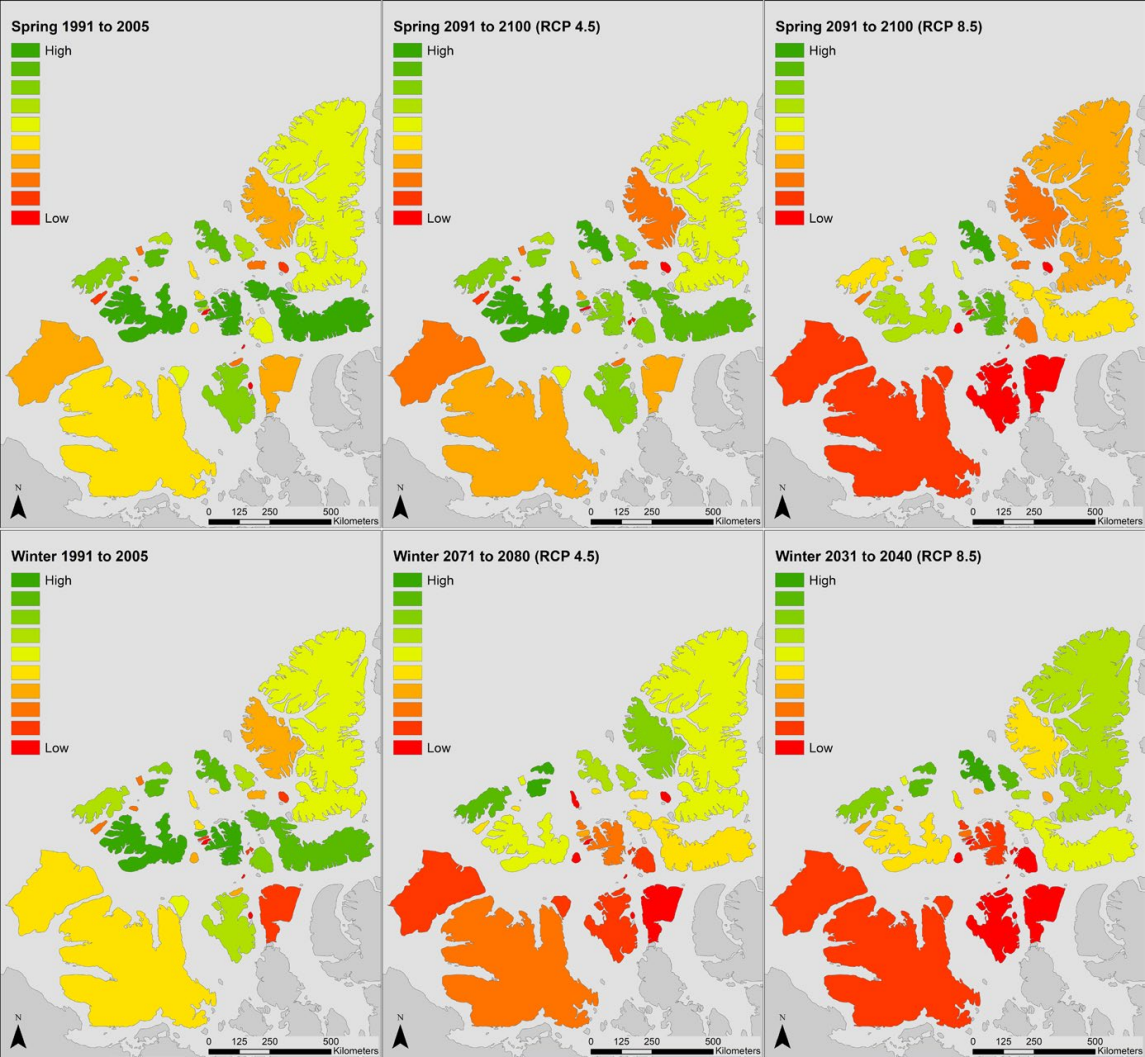
Current flow centrality across the study area in the Canadian Arctic Archipelago under the historical climate model (1991–2005), and selected decades from our RCP 4.5 and RCP 8.5 analyses. Current flow centrality represents the contribution made by an island to maintaining landscape connectivity across Peary caribou (*Rangifer tarandus pearyi*) range

We used two measures of current flow centrality to determine the importance of islands in the Canadian Arctic to maintaining Peary caribou connectivity: raw current flow centrality, and area‐corrected current flow centrality. The rankings of islands by current flow centrality are reported in tables in Appendix 1, and current flow centrality across the study area is shown in Figure 6.

Under the historical spring climate scenario, Bathurst Island had the highest centrality value followed by Melville, Devon, Mackenzie King, and Vanier Islands. When current flow centrality was corrected for island area, the importance of smaller islands in the archipelago (i.e., Massey, Little Cornwallis, King Christian, Vanier, and Emerald) became clear. Our projections indicated that by 2100 under the RCP 8.5 scenario, the most important islands for maintaining spring connectivity were Ellef Ringnes, Bathurst, Cameron, Vanier, King Christian, and Massey. When corrected for area, Massey, King Christian, Little Cornwallis, Cameron, and Vanier Islands had the highest centrality. Centrality rankings remained largely consistent over time in the RCP 4.5 scenario because of the smaller changes to landscape resistance (a notable exception is that by 2100 Ellef Ringnes Island holds the highest centrality and Bathurst Island ranks number 8, Appendix 1, Table A12).

In early‐winter, our model for the historical period from 1991 to 2005 indicated that Bathurst, Melville, Vanier, Devon, and Ellef Ringnes Islands had the highest centrality values. When corrected for area, Vanier, King Christian, Emerald, Brock, and Cameron Islands were most important for maintaining connectivity. By 2080 under the RCP 4.5 scenario, Borden, Mackenzie King, Prince Patrick, Axel Heiberg, and Amund Ringnes islands had the highest centrality. By 2040 under the RCP 8.5 scenario, Ellef Ringnes, Borden, Amund Ringnes, Mackenzie King, and Prince Patrick contributed most to maintaining connectivity. Although Table A23 (Appendix 1) provides rankings based on centrality for the RCP 4.5 2091–2100 and the RCP 8.5 2041–2050 periods, they should be interpreted cautiously because absolute current flow centrality values are very low due to the limited remaining connectivity and current flow across the landscape.

## 4 DISCUSSION

Other than the practical and financial difficulties associated with research in a vast and remote landscape (Mallory et al., 2018), Peary caribou provide a compelling example with which to study the effects of connectivity and fragmentation on a metapopulation that experiences regular random extirpations (or near‐extirpations) within local populations (Miller & Barry, 2009). Peary caribou exist in a naturally fragmented landscape, with strong seasonal variation in the level of connectivity. Decades of research on metapopulation dynamics have shown that connectivity between unstable local populations is necessary for long‐term metapopulation persistence (Fahrig & Merriam, 1985, 1994; Hanski, 1998; Kindlmann & Burel, 2008; Leimar & Norberg, 1997). Recent work on Peary caribou indicates that connectivity between local populations has declined over the past several decades, and is expected to further decrease with reduced Arctic sea ice cover (Jenkins et al., 2016). Here, we explored further the projected loss of connectivity for Peary caribou to identify those areas most important to maintaining linkages between populations.

In terms of connectivity loss, our results were broadly similar to those of Jenkins et al. (2016). Declines in sea ice coverage during our early‐winter period (November–December) exceeded those during our spring period (April–June), such that almost all connectivity was lost under both moderate (RCP 4.5) and high (RCP 8.5) GHG concentration scenarios for the winter period, while some connectivity remained in both spring scenarios (Figures 2, 4 and 5). Only the RCP 4.5 spring scenario retained connectivity between all Peary caribou local populations. All connections between populations were lost in winter by 2050 and 2100 in both the RCP 8.5 and 4.5 scenarios, respectively. Some early‐winter connections remained between WQEI and EQEI by 2040 (RCP 8.5) and 2080 (RCP 4.5), but may be unrealistic ecologically. For example, the connection between Borden Island and Axel Heiberg Island is approximately 315 km (Figure 5). Although Miller et al. (2005) reviewed a number of very long‐distance sea ice crossings by caribou and reindeer (including 340 and 380 km crossings), these types of movements are rarely documented and are unlikely to offset projected reductions in connectivity. Remaining connections between Ellesmere Island and Devon Island (approximately 14 km) and Axel Heiberg Island and Amund Ringnes Island (approximately 50 km) are more likely to be used. Across scenarios, islands inhabited by the WEQI local population remained the most connected, with islands in the Bathurst Island Complex (i.e., Bathurst, Cameron, Massey, and Vanier) and Melville and Prince Patrick Islands maintaining some measure of internal connectivity.

Islands in the Western Queen Elizabeth group are most important to maintaining connectivity across Peary caribou range (Appendix 1, Tables A12 and A23). The Bathurst Island complex, Melville and Prince Patrick Islands, and the Sverdrup Islands (specifically Amund Ringnes and Ellef Ringnes) appear critical to maintaining connectivity across the study area (Figure 6). Patches that lie toward the center of a study landscape generally have higher centrality scores than those at the periphery (Carroll et al., 2012; Dutta et al., 2016). Centrality scores in our study landscape typically follow this pattern, with islands in the center of the archipelago having high centrality (e.g., the Bathurst Island Complex), and those at the boundary having low centrality (e.g., Ellesmere and Victoria Islands). This is because to connect islands (or patches) near the edges, the shortest paths for current usually flow through the central islands. In many cases, it would thus be prudent to analyze connectivity some buffered distance beyond a study's area of interest to avoid introduction of these biases. However, for our study, the likelihood of any movement of Peary caribou not captured within the boundary of our analysis is very low. There have been some historical reports of Peary caribou crossing from Ellesmere Island to Greenland, but this would constitute a very small flow of individuals (COSEWIC, 2015). To the south, it is possible for Peary caribou to reach the mainland through Boothia Peninsula or by crossing south of Victoria Island, and indeed, there are some reports of local people observing Peary caribou in these areas. Again however, this behavior by Peary caribou does not seem to be common. Lastly, Peary caribou are not found on Baffin Island. We are thus confident the boundaries of our analysis are appropriate.

The importance of the WQEI for connectivity that we report here appears to be reflected in patterns of Peary caribou gene flow across the archipelago. McFarlane, Miller, Barry, and Wilson (2014) showed that genes flowed in a southern direction in the archipelago, from the WQEI population to the BV and PSB populations. No northward movement of genes was detected, thus highlighting the importance of WQEI as a source population for gene flow to more southern regions. Our models project that by 2100, unless atmospheric GHG concentrations are maintained at RCP 4.5 levels or below, all connectivity between WQEI and the BV and PSB populations will be lost during our spring and early‐winter periods (Figures 4 and 5). Although sea ice facilitated connectivity will remain during the late‐winter period (January to March), we consider the probability of long‐distance dispersals during this period to be lower. During the cold late‐winter period, caribou typically display more sedentary behavior. Movement rates are usually very low at this time of year (but not always, see Stuart‐Smith, Bradshaw, Boutin, Hebert, and Rippin (1997)) and movements to winter ranges often occur in the fall or early winter (Bergman, Schaefer, & Luttich, 2000; Brown et al., 1986; Fancy, Pank, Whitten, & Regelin, 1989; Ferguson & Elkie, 2004; Nagy, 2011). Movement data are sparse for Peary caribou, but analysis indicates seasonal ranges are smallest in winter (Miller & Barry, 2009) and most reports of long‐distance dispersals are in spring (Miller et al., 2005, 1977). In some areas of the archipelago, the majority of precipitation occurs in early winter, and loss of connectivity during this period could delay or increase the difficulty of escape from severe icing or snow conditions (Gunn & Dragon, 2002; Miller & Gunn, 2003b).

Sea ice coverage that reduces the dispersal and interisland movement ability of Peary caribou could have very serious consequences for long‐term metapopulation persistence. Given their small populations and the already limited connectivity, Peary caribou have reduced genetic diversity and heterozygosity compared to barren‐ground caribou (*R. t. groenlandicus*) on the Canadian mainland (Jenkins et al., 2018). Further loss of genetic variation could prove harmful, as the negative consequences of low genetic variability, such as reduced fitness and ability to adapt to environmental change, can be severe (Lacy, 1997; Lande, 1988; Petersen, Manseau, & Wilson, 2010). Reduced genetic variability warrants increased attention in the current context of climate change, where an improved ability to adapt to a changing environment associated with strong genetic variation across a population could greatly influence resilience and population persistence (Hoffmann & Sgró, 2011; Moritz & Agudo, 2013). However, it should be noted that to maintain genetic diversity, rates of immigration into populations need not be high. Low rates of immigration can contribute significantly to genetic diversity (Mills & Allendorf, 1996; Tallmon, Luikart, & Waples, 2004). For Peary caribou, migration rates from the WQEI to PSB population have been estimated at 16%–22% and from WQEI to the BV populations at 17% (McFarlane et al., 2014). Under moderate to low GHG concentration scenarios, it might be possible that enough rare dispersal events still occur during the shortened period of sea ice coverage to temper the loss of genetic variation.

A more immediate consequence of a longer ice‐free season is that recolonization of ranges from which caribou have been extirpated becomes more challenging. A specific example is that continued loss of seasonal connectivity means that the reestablishment of the PSB population will become increasingly improbable. As with the other populations across the archipelago, the abundance of PSB caribou has fluctuated over time. PSB caribou were reported to be at low numbers from the 1940s to early 1970s, with the population recovering to approximately 6,000 animals by 1980 (Gunn et al., 2006) before declining sharply and remaining at present levels of near extirpation (Anderson, 2016; Johnson et al., 2016). In order for the PSB area to be recolonized from WQEI (McFarlane et al., 2014), individuals are required to cross approximately 50–100 km of Barrow Strait. As the ice‐free season lengthens, opportunities for this crossing to occur will diminish.

Our results provide priority areas for future conservation and management efforts that target connectivity between Peary caribou populations. We reiterate the importance of islands with high centrality, such as the Bathurst Island complex and Melville Island, for maintaining connectivity across the entire archipelago. Connections across the Parry Channel, which separates the WQEI from the BV and PSB populations, have high conservation value despite their low centrality scores (Figures 3 and 4), and these links are vulnerable to being completely lost during important movement periods. The importance of the links between Bathurst, Melville, and Prince Patrick Islands (WQEI) and Banks, Victoria, Prince of Wales, and Somerset Islands (BV and PSB) comes from their critical role in facilitating the flow of genes between these three populations. While still important, higher‐latitude linkages between the WQEI and EQEI should have less conservation priority because they are more likely to persist over intermediate timescales. By considering area‐corrected centrality scores, our analysis highlights the importance of relatively small islands, including Little Cornwallis, King Christian, Borden, and Brock for landscape connectivity (Miller, 2002).

Typical conservation measures for enhancing connectivity are likely to involve undertakings such as protecting movement corridors, construction of wildlife crossing structures, and matrix restoration (Beier & Noss, 1998; Donald & Evans, 2006; Ng, Dole, Sauvajot, Riley, & Valone, 2004). However, the loss of connectivity across sea ice resulting from a warming climate presents a much different conservation problem for which these types of endeavors have limited applicability. What types of measures might then be effective? Of primary importance is the reduction of GHG emissions to limit further climate change. Across many studies, the projected negative effects of climate change on species and ecosystems are reduced under lower emissions scenarios (e.g., Garciá Molinos et al., 2016; Urban, 2015). From our findings, we observe that lower atmospheric GHG concentrations significantly improve the outlook for Peary caribou habitat connectivity. In a sense, this is good news for Peary caribou conservation, as GHG emission control and reduction is an important international subject with implications extending far beyond the ability of northern ungulates to move between islands. This means that global climate change mitigation efforts should have benefits for landscape connectivity in the Canadian Arctic Archipelago as a by‐product.

Habitat protection is another means by which conservation measures might be implemented. Although human and industrial activity in the Canadian High Arctic is currently low, extractive industries have existed in the region before (e.g., Polaris mine on Little Cornwallis Island, Bent Horn oil field on Cameron Island). Warmer temperatures and a longer shipping season are predicted to increase future levels of industrial development (Prowse et al., 2009). Regulators must carefully consider the potential effects to Peary caribou habitat connectivity that the loss of seemingly small areas of habitat could cause (i.e., pinch points, areas where landscape current flow is bottlenecked (Dutta et al., 2016)). For habitat protection, steps in this direction have already been taken with the establishment of Qausuittuq National Park on September 1, 2015. The park encompasses part of the Bathurst Island complex, including the northwestern portion of Bathurst Island and Vanier, Massey, Alexander, Helena, and several smaller islands in the complex. However, in their analysis of Peary caribou distribution in relation to the boundaries of Qausuittuq National Park, Poole, Gunn, Wierzchowski, and Anderson (2015) suggested mixed effectiveness of the park area in protecting Peary caribou habitat. Notably, the northeastern areas of Bathurst Island and the entirety of Cameron Island are excluded from the park due to their mineral and petroleum potential (Poole et al., 2015).

Finally, disruption to Peary caribou movement can occur through ice breaking. Ice breaking activities have been observed to disrupt caribou migrations across sea ice (Dumond, Sather, & Harmer, 2013), and while marine traffic in the High Arctic is currently limited, much like industrial activity it is expected to increase as sea ice coverage declines and the shipping season lengthens (Prowse et al., 2009). Beyond the climate‐driven lengthening of shipping windows, increasing development could bring with it pressure to artificially extend shipping seasons with ice breaking, or even year‐round shipping. These types of activities must be evaluated carefully and should not occur through connections between Peary caribou habitat during important movement periods in the spring and early winter.

### 4.1 Limitations of our analysis

Due to the nature of predictive climate modeling at a coarse scale, we must be cautious in interpreting our results. For example, although various climate models project Arctic sea ice decline, the specific spatial and temporal patterns of sea ice loss and formation can be variable across studies (e.g., Stroeve, Holland, Meier, Scambos, & Serreze, 2007). However, we suggest that the patterns we report should be generalizable across varying sea ice scenarios. In general, sea ice declines will occur more rapidly in southern regions of the study area and lead to loss of connectivity sooner at lower latitudes. Also, the Bathurst Island complex will have high centrality stemming from its central location within the distribution of Peary caribou. We reason that the many of the findings of our study should be robust and applicable beyond the particularities of our climate data and uncertainties therein.

## 5 CONCLUSIONS

Peary caribou are distributed at low densities over a vast area and a major threat to their persistence, loss of connectivity due to climate change, cannot be addressed directly by local land and resource managers. The extinction risk that climate change presents is difficult to curtail other than through coordinated international efforts to slow and limit GHG emissions (Bellard, Bertelsmeier, Leadley, Thuiller, & Courchamp, 2012; Maclean & Wilson, 2011; Thomas et al., 2004; Urban, 2015). However, we are hopeful that our research can help to highlight priority areas and actions that, when coupled with global reductions in GHG emissions, could help to mitigate the most negative consequences of connectivity loss.

## AUTHOR CONTRIBUTIONS

All authors contributed to the design of this study and writing of the manuscript. CDM performed all analysis.

## Data Availability

SIC projection data were taken from the output of the Canadian Regional Climate Model (CanRCM4) produced by the Canadian Centre for Climate Modelling and Analysis and were available from http://climate-modelling.canada.ca/climatemodeldata/canrcm/CanRCM4/index.shtml at the time of publication. Peary caribou probability of use data can be requested from ECCC. Please contact the authors for further information regarding data accessibility.

## APPENDIX 1 Current flow centrality rankings of islands in the Canadian Arctic Archipelago

**Table A1:** Spring current flow centrality rankings for all study islands in the Canadian Arctic Archipelago grouped by spring climate scenario

Island	Local population	Centrality					Area‐corrected centrality				
		Historical	RCP 4.5		RCP 8.5		Historical	RCP 4.5		RCP 8.5	
		1991–2005	2071–2080	2091–2100	2071–2080	2091–2100	1991–2005	2071–2080	2091–2100	2071–2080	2091–2100
Bathurst	WQEI	1	1	8	1	2	20	20	21	20	15
Melville	WQEI	2	2	2	2	8	23	22	23	22	18
Devon	WQEI	3	3	3	6	11	25	25	25	24	19
Mackenzie King	WQEI	4	5	5	5	7	14	14	14	13	10
Vanier	WQEI	5	4	4	4	4	4	4	2	4	5
Ellef Ringnes	WQEI	6	6	1	3	1	19	19	19	19	13
Prince of Wales	PSB	7	10	9	17	25	22	23	22	23	24
Prince Patrick	WQEI	8	7	7	12	12	21	21	20	21	16
Massey	WQEI	9	8	27	10	6	1	1	7	2	1
Borden	WQEI	10	9	11	9	9	11	11	11	10	9
Amund Ringnes	WQEI	11	11	6	8	13	17	16	15	15	14
Ellesmere	EQEI	12	12	12	15	14	28	28	28	27	23
Stefansson	BV	13	13	13	14	23	15	17	16	14	22
Cornwallis	WQEI	14	14	10	11	18	18	18	18	18	17
Byam Martin	WQEI	15	19	17	13	–	8	8	6	6	–
King Christian	WQEI	16	15	14	16	5	3	3	1	3	2
Cameron	WQEI	17	17	16	19	3	7	7	4	7	4
Little Cornwallis	WQEI	18	16	28	7	15	2	2	9	1	3
Lougheed	WQEI	19	18	15	18	10	10	9	8	9	7
Victoria	BV	20	20	19	24	22	29	29	29	29	26
Banks	BV	21	21	22	26	24	27	27	27	28	25
Somerset	PSB	22	22	18	25	–	24	24	24	25	–
Axel Heiberg	EQEI	23	23	21	21	19	26	26	26	26	21
Russell	PSB	24	26	23	27	26	9	10	10	12	20
Cornwall	WQEI	25	24	20	20	20	13	13	13	11	12
Brock	WQEI	26	25	24	22	16	6	6	5	8	8
Emerald	WQEI	27	27	25	23	17	5	5	3	5	6
Eglington	WQEI	28	28	26	28	21	12	12	12	17	11
Graham	EQEI	29	29	29	29	–	16	15	17	16	–

Note. Dashed lines indicate zero connectivity. Islands are listed in order of highest centrality from 1991 to 2005. Local populations: Western Queen Elizabeth Islands (WQEI), Eastern Queen Elizabeth Islands (EQEI), Prince of Wales/Somerset/Boothia (PSB), Banks/Victoria (BV)

**Table A2:** Winter current flow centrality rankings for all study islands in the Canadian Arctic Archipelago grouped by winter climate scenario

Island	Local population	Centrality					Area‐corrected centrality				
		Historical	RCP 4.5		RCP 8.5		Historical	RCP 4.5		RCP 8.5	
		1991–2005	*2071–2080	*2091–2100	*2031–2040	*2041–2050	1991–2005	2071–2080	2091–2100	2031–2040	2041–2050
Bathurst	WQEI	1	11	3	15	3	20	20	16	23	16
Melville	WQEI	2	8	2	12	2	22	17	18	19	18
Vanier	WQEI	3	10	1	14	1	1	10	3	13	3
Devon	WQEI	4	9	4	10	4	24	19	21	20	21
Ellef Ringnes	WQEI	5	6	5	1	5	19	11	19	11	19
Mackenzie King	WQEI	6	2	3	4	3	14	5	11	7	11
Cornwallis	WQEI	7	13	5	17	5	18	21	17	24	17
Borden	WQEI	8	1	4	2	4	11	3	10	4	10
Amund Ringnes	WQEI	9	5	5	3	5	15	7	15	8	15
Prince Patrick	WQEI	10	3	2	5	2	21	12	14	14	14
Prince of Wales	PSB	11	13	5	17	5	23	23	22	25	22
Ellesmere	EQEI	12	8	3	6	3	28	22	24	22	24
Stefansson	BV	13	12	4	16	4	17	18	12	21	12
King Christian	WQEI	14	9	5	13	5	2	4	8	5	8
Victoria	BV	15	11	3	15	3	29	25	25	27	25
Banks	BV	16	12	4	16	4	27	24	23	26	23
Lougheed	WQEI	17	–	–	9	–	10	–	–	2	–
Cameron	WQEI	18	11	3	15	3	5	13	4	15	4
Byam Martin	WQEI	19	–	–	–	–	9	–	–	–	–
Cornwall	WQEI	20	9	5	7	5	13	9	13	6	13
Russell	PSB	21	13	5	17	5	6	16	9	17	9
Axel Heiberg	EQEI	22	4	4	11	4	26	15	20	18	20
Brock	WQEI	23	7	4	8	4	4	1	5	1	5
Emerald	WQEI	24	9	3	13	3	3	2	2	3	2
Eglington	WQEI	25	9	3	13	3	12	6	7	10	7
Somerset	PSB	26	–	–	–	–	25	–	–	–	–
Massey	WQEI	27	11	3	13	3	7	–	1	9	1
Little Cornwallis	WQEI	28	13	5	15	5	8	14	6	12	6
Graham	EQEI	29	–	–	17	–	16	8	–	16	–

Note. Dashed lines indicate zero connectivity. Islands are listed in order of highest centrality from 1991 to 2005. Years with an asterisk indicate that some islands during this period have equivalent centrality rank. Local populations: Western Queen Elizabeth Islands (WQEI), Eastern Queen Elizabeth Islands (EQEI), Prince of Wales/Somerset/Boothia (PSB), Banks/Victoria (BV)
